# Design of Narrow Discrete Distances of Dual-/Triple-Band Terahertz Metamaterial Absorbers

**DOI:** 10.1186/s11671-019-2876-3

**Published:** 2019-02-22

**Authors:** Ben-Xin Wang, Chao Tang, Qingshan Niu, Yuanhao He, Tao Chen

**Affiliations:** 1 School of Science, Jiangnan University, Wuxi, 214122 China; 2 School of Mechanical Engineering, Hubei University of Technology, Wuhan, 430068 China

**Keywords:** Metamaterial, Perfect absorber, Terahertz, Narrow discrete distance

## Abstract

Various kinds of structure designs have been proposed to achieve the multiple-band metamaterial absorbers. However, the discrete distance of adjacent frequencies of multiple absorbers is considerably large, which will inevitably overlook a large amount of information hidden in the off-resonance absorption areas. Herein, a narrow discrete distance of dual-band terahertz absorber based on two pairs of an Au strip/dielectric layer backed by Au film is designed. Two nearly 100% absorptivities of resonance peaks having the discrete distance of only 0.30 THz are realized. The relative discrete distance of the device is 13.33%, and this value can be adjusted via the length change of an Au strip. Furthermore, we present two narrow discrete distances of a triple-band absorber through stacking one more pair of an Au strip and dielectric layer. Results prove that two discrete distances of only 0.14 THz and 0.17 THz in adjacent absorption modes of the first two and the last two are achieved, respectively; the relative discrete distances of them are respectively 6.57% and 7.22%, which are far from previous reports. Narrow discrete distances (or low values of relative discrete distance) of the multiple-band absorbers have a large number of applications in the investigation of some hidden information in very near frequencies.

## Introduction

Metamaterial perfect absorbers (abbreviated as MPAs) as an important part of optical absorption devices have attracted considerable research activities because they possess many advantages over others, such as ~ 100% absorption, ultra-thin thickness of dielectric layer, narrow absorption bandwidth, and freedom design of pattern structure [1–12]. The first design concept of MPA [13], consisted of a sandwich structure of an electric-ring resonator, insulation dielectric layer, and metallic strip, was presented by a research group from Boston College in the year of 2008. A resonance peak with an absorption rate of higher than 88% at a frequency of 11.5 GHz can be experimentally obtained. The dielectric thickness of the device is only about 1/35 of the absorption wavelength, which is far less than previous absorption devices. The MPA with these features can be potentially used in bolometer, sensing, detection, and imaging. However, a narrow acceptance angle, polarization sensitivity, and single-band absorption response are the disadvantages of the presented MPAs.

To overcome these issues [14–24], many works have been suggested to develop the wide-angle, polarization-insensitive, multiple-band and even broadband MPAs through reasonable optimization of structure designs. For example, a wide-angle optical MPA based on 1-dimensional stack array of a resonance structure was suggested in ref. [18]. Nested metallic ring resonators were demonstrated to get the multiple-band resonance absorption [19–23]. In the development and research process of absorption devices, multiple-band MPAs, which can be used for some of dangerous goods (dynamite, detonator, and alcohol) detection, spectroscopic imaging (various types of controlled knives), sensing, and selective bolometer, have received tremendous attention [19–30].

Generally speaking, three kinds of methods can be used to achieve the multiple-band MPAs. The first method, commonly referred to as a coplanar construction method, is formed by multiple different sizes of resonators in a super-unit structure [19–26]. The second is called the vertical stacked method, consisted of alternate stacks of multiple discrete dimensions of elements [27–30]. The third is the combination of the first two methods [31, 32]. Although these approaches can flourish and develop the multiple-band MPAs, the discrete distances of the resonance frequencies of adjacent absorption peaks are quite large. A large discrete distance in two adjacent frequencies will inevitably overlook a lot of information hidden in the off-resonance areas, i.e., the discrete areas. In order to avoid the loss of information, therefore, the large discrete distance of multiple-band MPAs should be overcome. Although the discrete distances of multiple-band MPAs can be reduced via suitable structure optimization, the off-resonance absorption areas of them are relatively large (greater than 60%), they should be called the broadband MPAs [33–40], not the multiple-band MPAs. As everyone knows, multiple-band and broadband MPAs are essentially different in their applications. Therefore, it is necessary to ensure low absorption rates (less than 60%) of off-resonance areas in the optimization for reduction of discrete distances.

In fact, a relative discrete distance should be more meaningful than a discrete distance because it can reflect the true information of two adjacent frequencies. The relative discrete distance (△) of two adjacent peaks can be defined as △ = 2(*f*_2_ − *f*_1_)/(*f*_1_ + *f*_2_), where *f*_1_ and *f*_2_ are the frequencies of two neighboring peaks. To guarantee △ > 0, the frequency of *f*_2_ should be higher than that of *f*_1_. According to this definition, the minimum △ values of previous multiple-band MPAs are typically no less than 50% [19–30], which are far from satisfactory to explore and investigate the hidden messages in the areas of adjacent frequencies. It is very reasonable, therefore, to develop multiple-band MPAs with very near frequencies or low values of △.

In this paper, we present the low △ value of dual-band terahertz MPA formed by dual-layer stack of Au strips and insulation dielectric layers backed by a continuous Au plane. Two nearly perfect absorption peaks with discrete distance of only 0.30 THz are obtained. The △ value of device is 13.33%, which is only 1/4 of the previous minimum value of MPAs, and the △ value can be tuned through dimension change of the Au strips. Its △ value can be reduced to only 6.45%, which is far less than that of previous MPAs. A narrow discrete distance or low △ value of the dual-band MPA is caused by the ultra-narrow bandwidth of each resonance band. We furthermore present two low △ values of triple-band MPA through stacking one more Au strip. Two narrow discrete distances of only 0.14 THz and 0.17 THz in three nearly perfect absorption peaks can be realized; the △ values of adjacent frequencies of triple-band MPAs are respectively 6.57% and 7.22%, which are both smaller than that of previous works. Low △ values of these MPAs can find a number of applications in the study of some implicit information in the areas of off-resonance absorption.

## Methods/Experimental

**Fig. 1:**
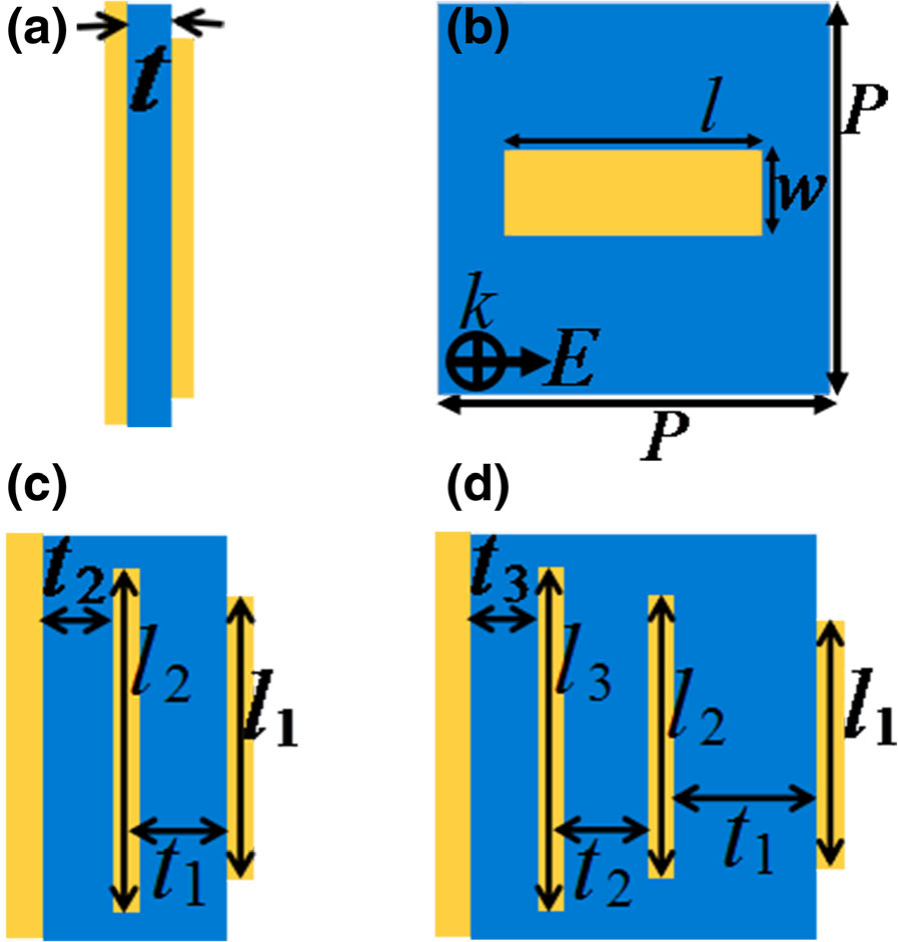
Side views of the single-band, dual-band, and triple-band MPAs are respectively presented in **a**, **c**, and **d**; **b** gives the top view of the Au strip resonator

In general, the bandwidth (refers to FWHM, full wave at half maximum) of single-band MPA is relatively wide, which can reach 20% of center resonance frequency, because of strong resonance response of metamaterials. The combination of these single-band peaks to form multiple-band MPAs inevitably possesses large values of discrete distance or △. This is why the previous multiple-band MPAs have large △ values. The key to obtain the low values of △ is to design the narrow bandwidth of single-band MPAs. Herein, we first design this kind of single-band MPA. A common sandwich structure formed by an Au resonator and a certain thickness of dielectric material backed by an Au mirror is employed to achieve the single-band absorption, as illustrated in Fig. 1a. The Au resonator is a rectangular strip structure, see Fig. 1b. It has the length of *l* = 39 μm, width of *w* = 8 μm, thickness of 0.4 μm, and conductivity of 4.09 × 10^7^ S/m. The MPA has a unit period of *P* = 60 μm. The dielectric slab has a thickness of *t* = 2 μm and dielectric constant of 3(1 + *i*0.001).

To present the resonance performance of the suggested device and explain the physical mechanism involved, we performed numerical calculations using the commercial simulation software, FDTD Solutions, which is based on the finite-difference time-domain algorithm. In the computing process, periodic boundary conditions are utilized in both directions of *x*- and *y*-axes to characterize the periodic arrangement of the unit cell, while perfectly matched layers are employed along the direction of the *z*-axis (i.e., the light propagation direction) to eliminate the unnecessary scattering. The absorption (*A*) of the device can be given by *A* = 1 – *T* − *R*, where *T* and *R* are the transmission and reflection of the metamaterial absorber, respectively. Because the thickness of the bottom metallic film is greater than the skin depth of the incident light, the transmission *T* of the metamaterial absorber is equal to zero. As a result, the absorption *A* can be simplified to *A* = 1 − *R*. The suggested device can have 100% absorption when the reflection *R* is completely suppressed.

## Results and Discussion

**Fig. 2:**
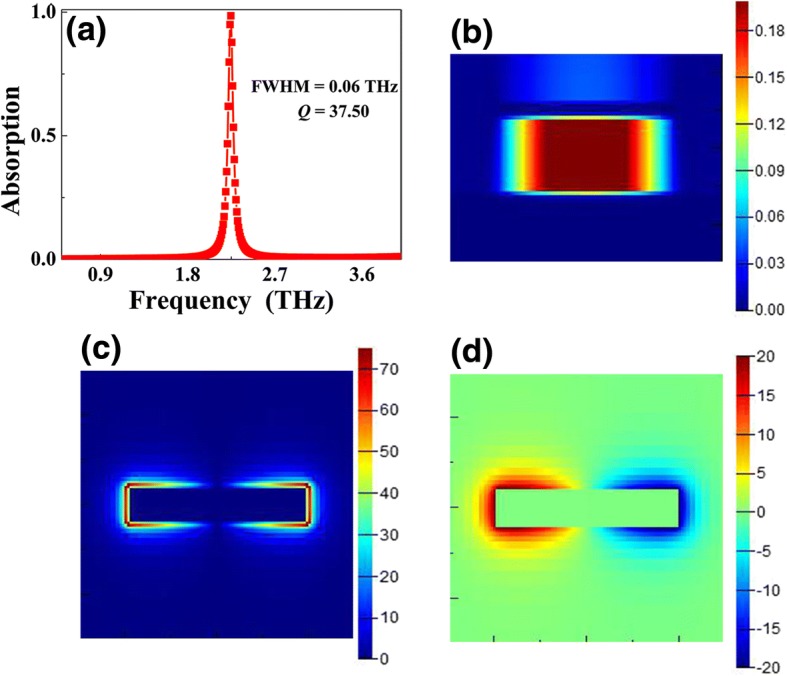
Absorption curve of the single-band MPA under plane wave irradiation is provided in **a**; **b**, **c**, and **d** give the field distributions of the |*H*y|, |*E*|, and *E*z at a peak of 2.25 THz, respectively

The absorption curve of single-band MPA under plane wave irradiation is demonstrated in Fig. 2a; ~ 100% absorption of a single resonance peak at a frequency of 2.25 THz is obtained. The bandwidth of the device is 0.06 THz, which is only 2.67% of the center resonance frequency and is about 1/8 of a previous single-band MPA [1–13]. Additionally, the *Q* (defined as resonance frequency divided by bandwidth) value of the device can be up to 37.50. The ultra-narrow bandwidth (or high *Q* value) of MPA not only contributes to the applications of device itself, but also helps to the design of low △ value of multiple-band MPAs. Figure 2b, c, and d provide the field distributions of the resonance peak. As shown, its magnetic field (|*H*y|) in Fig. 2b is mostly concentrated in an insulation dielectric layer of MPA, and strong electric field enhancement can be observed at both sides of the Au resonator along the long axis (see Fig. 2c, d). These field distribution features indicate that the large light absorption of narrow bandwidth of MPA is due to the magnetic resonance [1–4].

We next explore whether the combination of these narrow bandwidth of MPAs has the ability to realize the low △ value of multiple-band MPAs. A vertically stacked design concept, as a kind of frequently used method, is employed to obtain the multiple-band MPAs. An example of the simplest kind is the case of dual-band absorption. Figure 1c gives the side-view of the structure model of the dual-band absorption. As shown, two layers of metallic strip resonators and insulation dielectric slabs are alternately stacked on a metallic ground plane. The lengths of two Au strips are respectively *l*_1_ = 36 μm and *l*_2_ = 39 μm; the widths of them are fixed as *w* = 8 μm. The thicknesses of dielectric slabs are *t*_1_ = 1.4 μm and *t*_2_ = 2 μm. Other parameters of the dual-band MPA, including unit period, dielectric constant of the slab, thickness, and conductivity of Au strips, are the same as those of the single-band MPA.

**Fig. 3:**
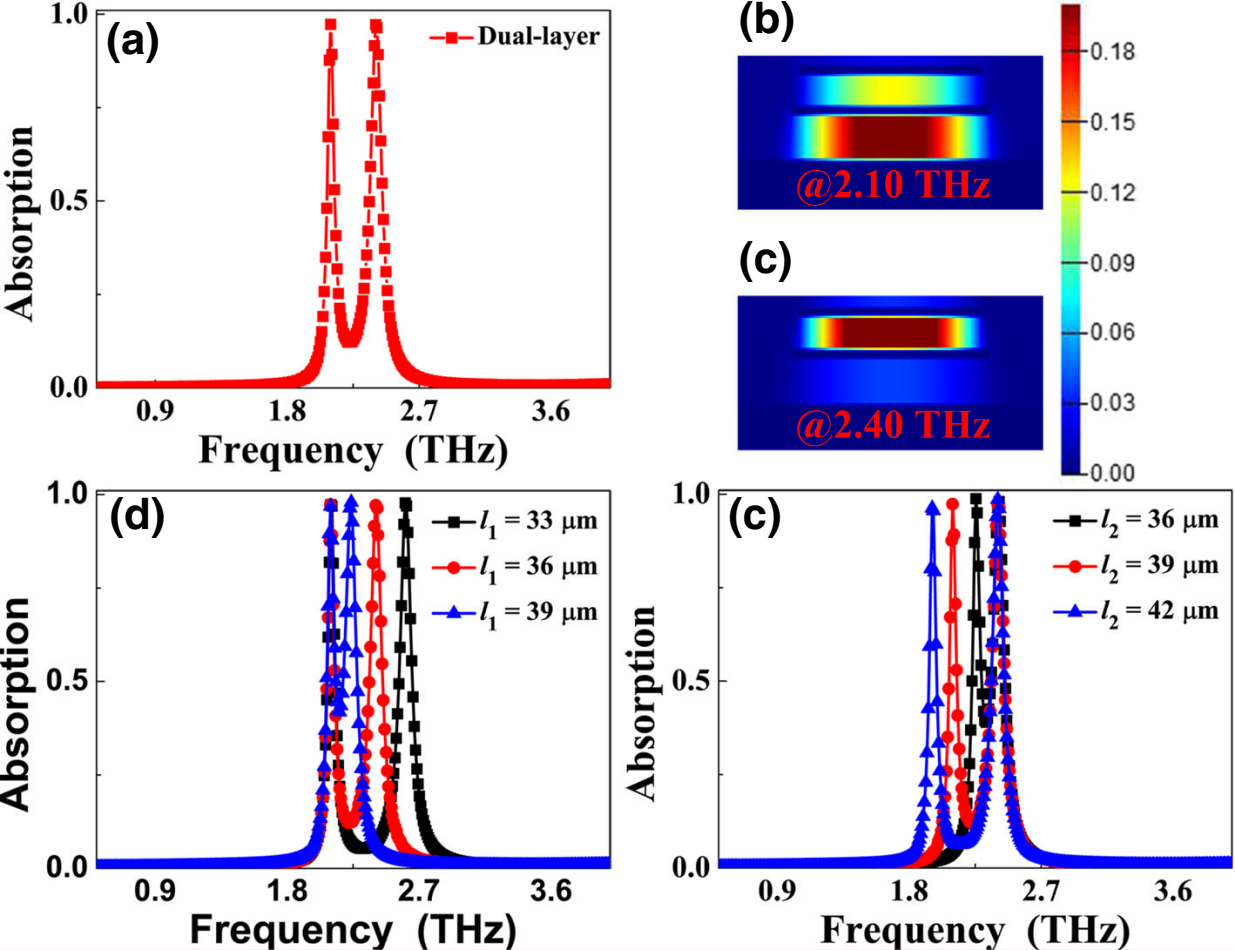
Absorption curve of dual-band MPA under plane wave irradiation is presented in **a**; **b** and **c** provide the |*H*y| field distributions of the first and the second modes of dual-band MPA, respectively. Absorption curves of dual-band MPA under different lengths of *l*_1_ and *l*_2_ are demonstrated in **d** and **e**, respectively

The absorption curve of the dual-band MPA under plane wave irradiation is illustrated in Fig. 3a. Different from the case of the single-band MPA in Fig. 2a, two resonance peaks with ~ 100% absorption rates at frequencies of 2.10 THz and 2.40 THz are achieved. The bandwidths of the two peaks are respectively 0.05 THz and 0.09 THz, which are only 2.00% and 3.75% of corresponding resonance frequencies, respectively. The *Q* values of the two peaks are 42.00 and 26.67, respectively. Additionally, the off-resonance absorption of the two peaks is very low, less than 12%. These features show that the two peaks having narrow bandwidths can be clearly distinguished. It is important that the discrete distance of the two peaks is only 0.30 THz, and its △ is 13.33%, which is smaller than that of previous works [19–30]. The low △ value of dual-band MPA is promising in many areas of engineering and technology. The resonance mechanisms of the two absorption peaks can be gained by analyzing their magnetic fields |*H*y|. The field |*H*y| for the first peak is mostly focused on the second dielectric slab of the dual-band MPA, while the field in the first dielectric layer has a very small percentage (see Fig. 3b). The characteristics of field distribution prove that the first absorption mode is attributed to magnetic resonance of the second dielectric layer, or the first peak frequency is caused by metallic strip length *l*_2_ (see Fig. 3e). Different from the case of the first resonance mode, the |*H*y| field of the second mode is primarily distributed in the first layer of dielectric slab (see Fig. 3c), which indicates that this mode is derived from magnetic resonance of the first dielectric slab, or its resonance frequency can be tuned via varying the size of strip length *l*_1_ (see Fig. 3d), and thus tune the △ value of the dual-band MPA.

The △ values of the dual-band MPA can be adjusted through changing the sizes of Au strips because the frequencies of the two modes mainly depend on the corresponding sizes of strips. For example, for length *l*_1_ change of the first layer of Au strip (see Fig. 3d), the frequency of the second mode gradually decreases with the increase of *l*_1_, while the frequency shift of the first mode can be neglected because its size is fixed. The discrete distances of the two peaks are varied because of the frequency shift of the second mode. More concretely, the discrete distances can be decreased from 0.41 THz in *l*_1_ = 33 μm to 0.30 THz in *l*_1_ = 36 μm and 0.23 THz in *l*_1_ = 39 μm. The △ values of the dual-band MPA can also be decreased from 17.41% in *l*_1_ = 33 μm to 13.33% in *l*_1_ = 36 μm and 10.38% in *l*_1_ = 39 μm. That is to say, the strip length *l*_1_ change can decrease the discrete distances and the △ values. Similarly, the strip length *l*_2_ change only affects its corresponding resonance frequency, i.e., the first resonance mode, see Fig. 3e. The discrete distances and △ values of dual-band MPA are both decreased with *l*_2_ decrease because the first mode frequency with the decrease of *l*_2_ is gradually close to the second absorption peaks, as shown in Fig. 3e. When *l*_2_ = 36 μm, the discrete distance has the smallest value, which is 0.15 THz. At this time, its △ value is only 6.45%, which is smaller than that of previous reports. These results prove that the discrete distances (or △ values) of dual-band MPA can be controlled to meet the requirements of different applications through tuning the sizes of Au strips.

**Fig. 4:**
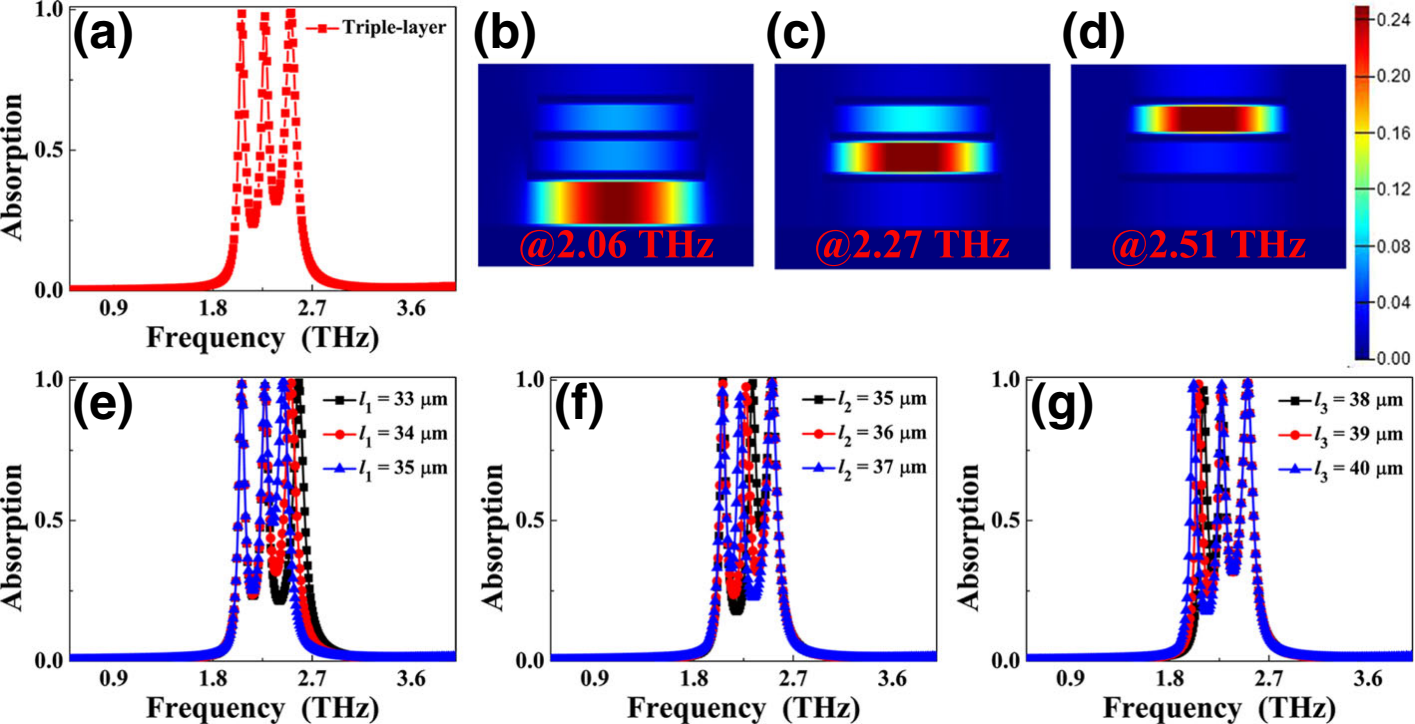
Absorption curve of triple-band MPA under plane wave irradiation is given in **a**; **b**, **c**, and **d** show the |*H*y| field distributions of the first, the second, and the third modes of the triple-band MPA, respectively. Absorption curves of the triple-band MPA under different lengths of *l*_1_, *l*_2_, and *l*_3_ are demonstrated in **e**, **f**, and **g**, respectively

We further investigate whether the stack of one more Au strip (i.e., triple-layer structure) can achieve two low △ values of triple-band MPAs. Figure 1d presents a side view of a triple-layer structure model of MPA, which is consisted of three pairs of Au strip/dielectric slab on top of an Au mirror. The Au strips have lengths of *l*_1_ = 34 μm, *l*_2_ = 36 μm, and *l*_3_ = 39 μm. The dielectric slabs have thicknesses of *t*_1_ = 1.2 μm, *t*_2_ = 1.4 μm, and *t*_3_ = 2.8 μm, respectively. The widths of Au strips are all *w* = 8 μm. Other parameters of the triple-layer MPA are the same as designed above. The absorption curve of the triple-layer MPA under plane wave irradiation is shown in Fig. 4a. Three discrete peaks having ~ 100% absorption rates at frequencies of 2.06 THz, 2.27 THz, and 2.51 THz can be found. The discrete distances of adjacent peaks in resonance modes of the first two and the last two are respectively 0.21 THz and 0.24 THz. The △ values of the modes of the first two and the last two are 9.70% and 10.04%, respectively, which are both less than that the values of multiple-band MPAs. In addition to narrow discrete distances, the absorption rates in off-resonance areas of the triple-band MPA are relatively low, no more than 32% (see Fig. 4a). It is shown that the three very near peaks can be clearly identified and can be used for sensing, detection, imaging, and application to other tasks. The |*H*y| field distributions of the three absorption peaks are provided to analyze the resonance mechanism of the triple-band MPA. As shown in Fig. 4, the |*H*y| field distributions of the first, the second, and the third mode of the triple-band MPA can be mainly found in the dielectric layers of *t*_3_, *t*_2_, and *t*_1_, respectively, while the fields in other dielectric layers are negligible. For example, for the first mode in Fig. 4b, the fields in dielectric layers of *t*_2_ and *t*_1_ can be neglected, and the fields in dielectric layers of *t*_2_ and *t*_3_ are negligible for the third mode in Fig. 4d. These distribution features state clearly that the three absorption peaks are all caused by magnetic resonances. More specifically, the first, the second, and the third modes are attributed to magnetic resonances of the third dielectric layer *t*_3_, the second dielectric layer *t*_2_, and the first dielectric layer *t*_1_, respectively, or the frequencies of the first, the second, and the third mode are dependent on Au strip lengths of *l*_3_, *l*_2_, and *l*_1_, respectively.

The △ values of the triple-band MPA can be controlled through adjusting the Au strip lengths. Figure 4e gives the absorption curves of the triple-band MPA in different cases of length *l*_1_. As you can see, the *l*_1_ change mainly affects the third mode frequency, while the frequency shifts of the first two modes are negligible, which are consistent with the theoretical prediction. Due to the frequency variation of the third mode, we can tune the △ value of the last two modes of the triple-band MPA. The △ values of the last two modes can be tuned from 12.66% in *l*_1_ = 33 μm to 10.04% in *l*_1_ = 34 μm, and 7.22% in *l*_1_ = 35 μm. The △ value of the first two modes can also be controlled by adjusting length *l*_3_ (see Fig. 4g). The minimum discrete distance of the first two modes is 0.16 THz for *l*_3_ = 38 μm, and its △ value is 7.31%. Furthermore, we can tune the △ values of the first two and last two modes via scaling the length *l*_2_, i.e., the frequency of the second mode (see Fig. 4f). Remarkably, the △ value changes of the first two and last two modes are mutual restriction because we only change the frequency of the second mode. For example, for *l*_1_ = 37 μm (see the blue line in Fig. 4f), the discrete distance of the first two modes has the minimum value of 0.16 THz, while maximum value of 0.29 THz for last two modes can be obtained.

## Conclusion

In conclusion, a narrow discrete distance of the dual-band terahertz MPA consisted of two pairs of Au strip/dielectric slab backed by an Au film is presented. Two ~ 100% absorption rates of resonance peaks having the discrete distance of 0.30 THz are realized, and the △ of the dual-band MPA is 13.33%. The mechanism of dual-band absorption is caused by superposition effects of two different frequencies of magnetic resonances. We can further adjust △ values of the dual-band MPA through employing different lengths of Au strips. The △ value can be decreased to only 6.45%, which is much lower than that of previous results. Moreover, two narrow discrete distances of the triple-band MPA are demonstrated by stacking one more pair of strip/dielectric. Three ~ 100% absorptivities of resonance peaks with discrete distances of 0.21THz and 0.24 THz are achieved. The △ values of two adjacent frequencies (that are the modes of first two and the last two) are respectively 9.70% and 10.04%. Similar to the case of dual-band absorption, the triple-band MPA also has the ability to tune the △ value of adjacent frequencies by controlling the lengths of Au strips. Narrow discrete distances or low △ values of multiple-band MPAs are promising in many areas, such as investigation of some implicit information in two very near frequencies.

## Abbreviations

FWHM: Full wave at half maximum
MPAs: Metamaterial perfect absorbers
Q: Quality factor
